# Routine Bone Imaging for Metastatic Renal Cell Carcinoma: Is it Time?

**DOI:** 10.15586/jkcvhl.v8i4.207

**Published:** 2021-10-14

**Authors:** Mamta Parikh

**Affiliations:** University of California Davis Comprehensive Cancer Center, Sacramento, CA, USA

Current guidelines by the National Comprehensive Cancer Network recommend that, in addition to routine computed tomography (CT) imaging, bone imaging and brain magnetic resonance imaging (MRI) should be obtained when clinically indicated. In this issue of the *Journal of Kidney Cancer and VHL*, a systematic literature review of clinical trials of metastatic renal cell carcinoma (mRCC) patients evaluates the incidence of osseous, lymph node, and lung metastases (1). In particular, the analysis by Lin et al focuses on the changes in incidence over time. The study finds that the incidence of bone, lymph node, and lung metastases has increased over time. This increase is significant in osseous metastases specifically. These results lead to two provocative questions. First, why have osseous metastases increased in incidence over time? Second, does this finding warrant a more aggressive and uniform approach to imaging to identify osseous metastases sooner?

The cause of an increased incidence of osseous metastases overtime may be multifactorial. For example, an increased incidence of kidney cancer has been appreciated for some time and is partly because of advances in and increased use of imaging (2). This may, in part, explain the increased incidence of osseous metastases over time. One limitation of the current analysis is that the authors could not delve into granular details of imaging requirements in the compiled trials and their change over time. Increased incorporation of bone imaging into trial protocols may have resulted in higher detection of osseous metastases.

However, just as the increased incidence of kidney cancer likely cannot be explained by increased use of imaging alone, similarly, the natural history of kidney cancer must be considered when examining this increased incidence of bone metastases. In particular, the evolving treatment paradigms of mRCC have led to dramatic extensions in survival. For example, consider that the SWOG-8949 trial of patients treated with interferon alfa-2b and cytoreductive nephrectomy demonstrated a median overall survival (OS) of 11.1 months (3). In contrast, even with extended follow-up from the more recent KEYNOTE-426 study, the median OS of patients treated with the combination of pembrolizumab plus axitinib has still not been reached, whereas the median OS of patients treated with sunitinib was 35.7 months (4). Thus, an increased incidence of bone metastases may be a result of extended longevity leading to development of additional metastatic sites. Interestingly, the current analysis did evaluate differences in the incidence of bone metastases in the first-line setting (28%) versus in patients being treated in the second-line setting or beyond (29%). So, this study has not demonstrated that osseous metastases are late-stage sequelae of mRCC.

Another limitation of the literature review is that the outcomes were not evaluated concerning osseous metastases. Some studies have suggested that patients with bone metastases have worse results. An analysis of the International Metastatic Renal Cell Carcinoma Database Consortium during the era of vascular endothelial growth factor tyrosine kinase inhibitor (VEGF-TKI) therapy demonstrated a median survival of 14.9 months for patients with bone metastases, compared with 25.1 months for those without bone metastases (5).

Demonstration of an increased incidence of bone metastases over time is not independently a justification for the adoption of routine bone imaging in all patients with mRCC. When determining whether a test should be routinely and uniformly utilized, the application of its results must also be considered. In the case of osseous metastases, one must consider the benefits of early intervention in asymptomatic patients or if therapies are specifically beneficial to patients with osseous metastases. Bone-targeted therapies like zoledronic acid and denosumab have shown demonstrated benefits in delaying skeletal-related events in mRCC patients in small studies or subset analyses. Their gain has not been significant in the current era since the use of VEGF-TKIs (6). Though bone-targeted therapies are still routinely used in patients with mRCC, their use alone is not currently a justification for routine bone imaging.

Whether mRCC patients with osseous metastases may benefit from specific therapies remains an area of interest. Cabozantinib could potentially alter the bone microenvironment, and as a result, been studied in patients with mRCC who have osseous metastases. The Phase III METEOR study evaluated and demonstrated a clinical benefit of cabozantinib compared with everolimus in patients with refractory mRCC (7, 8). The protocol required routine bone imaging before enrollment and during treatment. Subgroup analysis was performed on this study of patients with osseous metastases at baseline before treatment, demonstrated superior progression free survival, OS, and objective response rate in patients treated with cabozantinib. As the landscape of mRCC continues to evolve, findings such as these become pivotal in determining the appropriate sequencing of therapies in the second line and beyond setting. A current cooperative group study (RadiCaL) is evaluating the combination of cabozantinib with or without Radium-223 dichloride in mRCC patients who have osseous metastases (NCT04071223). In the first-line setting, insufficient data are comparing current standard of care treatments that combine immune checkpoint inhibitors with VEGF TKIs to determine if a cabozantinib-containing combination is beneficial in treatment-naïve mRCC patients with osseous metastases.

While it may be premature to incorporate routine bone imaging in the treatment of mRCC patients based on the results of this systematic literature review, including routine bone imaging in clinical trials may become increasingly important for enhanced determination of sequencing or choice of therapies. Studies designed to evaluate patients with bone metastases specifically may allow for data that may justify the use of routine bone imaging in the future.
